# Children With Autism Spectrum Disorder and Their Mothers Share Abnormal Expression of Selected Endogenous Retroviruses Families and Cytokines

**DOI:** 10.3389/fimmu.2019.02244

**Published:** 2019-09-26

**Authors:** Emanuela Balestrieri, Chiara Cipriani, Claudia Matteucci, Arianna Benvenuto, Antonella Coniglio, Ayele Argaw-Denboba, Nicola Toschi, Ilaria Bucci, Martino Tony Miele, Sandro Grelli, Paolo Curatolo, Paola Sinibaldi-Vallebona

**Affiliations:** ^1^Department of Experimental Medicine, University of Rome Tor Vergata, Rome, Italy; ^2^Child Neurology and Psychiatry Unit, Systems Medicine Department, University Hospital Tor Vergata, Rome, Italy; ^3^European Molecular Biology Laboratory (EMBL), Rome, Italy; ^4^Department of Biomedicine and Prevention, University of Rome Tor Vergata, Rome, Italy; ^5^Athinoula A. Martinos Center for Biomedical Imaging, Harvard Medical School, Boston, MA, United States; ^6^Institute of Translational Pharmacology, National Research Council, Rome, Italy

**Keywords:** HERVs, HEMO, cytokines, autism spectrum disorder, biomarker, mother-child association, gene expression

## Abstract

The Autism Spectrum Disorder (ASD) is a heterogeneous group of neurodevelopmental disorders, only clinically diagnosed since the lack of reliable biomarkers. Autism etiology is probably attributable to the combination of genetic vulnerability and environmental factors, and recently, maternal immune activation has been linked to derailed neurodevelopment, resulting in ASD in the offspring. Human endogenous retroviruses (HERVs) are relics of ancestral infections, stably integrated in the human DNA. Given the HERV persistence in the genome, some of HERVs have been co-opted for physiological functions during evolution, while their reactivation has been associated with several pathological conditions, including cancer, autoimmune, and neurological and psychiatric disorders. Particularly, due to their intrinsic responsiveness to external stimuli, HERVs can modulate the host immune response and in turn HERVs can be activated by the immune effectors. In previous works we demonstrated high expression levels of HERV-H in blood of autistic patients, closely related with the severity of the disease. Moreover, in a preclinical ASD model we proved changes of expression of several ERV families and cytokines from the intrauterine life to the adulthood, and across generations *via* maternal lineage. Here we analyzed the expression of HEMO and of selected HERVs and cytokines in blood from ASD patients and their parents and corresponding healthy controls, to look for a common molecular trait within family members. ASD patients and their mothers share altered expression of HERV-H and HEMO and of cytokines such as TNF-α, IFN-γ, IL-10. The multivariate regression models showed a mother-child association by HEMO activity and demonstrated in children and mothers an association between HERV-H and HEMO expression and, only in mothers, between HEMO, and TNF-α expression. Furthermore, high diagnostic performance for HERV-H and HEMO was found, suggesting their potential application for the identification of ASD children and their mothers. The present data support the involvement of HERVs in ASD and suggest HERVs and cytokines as ASD-associated traits. Since ASD is a heterogeneous group of neurodevelopmental disorders, a single determinant alone could be not enough to account for the complexity, and HERV/cytokines expression could be considered in a set of biomarkers, easily detectable in blood, and potentially useful for an early diagnosis.

## Introduction

The Autism Spectrum Disorder (ASD) is an early onset, complex and heterogeneous neurodevelopmental disorder, affecting around 1% of the population (1). ASD diagnosis is based on clinical observation and neuropsychological assessment of the core deficits in social communication and restricted and repetitive patterns of behavior, lacking reliable biomarkers for clinical use (2). To date, ASD etiology remains unknown, likely resulting from a combination of genetic vulnerability and environmental factors (2). Numerous ASD-risk genes have been identified and copy number variations, polymorphisms and *de novo* mutations might be also involved in the ASD pathogenesis (3, 4). Nevertheless, the heterogeneity of clinical/biological phenotypes and large difference in concordance rate in ASD dizygotic/monozygotic twin pairs suggest that the environmental factors play a crucial role during the critical phase of central nervous system development (5). Specifically, the vulnerability of offspring with ASD is related to the pre-peri and early postnatal exposure to environmental factors ranging from pollutants, xenobiotics, or chemical agents like valproate, to pathogens, and maternal immune activation (6).

In the last two decades, compelling evidences involve human endogenous retroviruses (HERVs) activity in complex diseases including cancer, autoimmune, neurological, and psychiatric disorders such as schizophrenia, attention deficit/hyperactivity disorder (ADHD), and ASD (7–12, 58). HERVs are relics of ancestral germline infections by exogenous retroviruses, resulting in proviruses transmitted to progeny in a Mendelian fashion and stably integrated in the human DNA, in which they constitute about 8% of the genome (13–15). HERVs are grouped into several families, according to the sequence homology, and the time in which their integration occurred (16). Recently, a new envelope gene, named HEMO [human endogenous MER34 (medium-reiteration-frequency-family-34) ORF], encoding a full-length retroviral protein, produced by the *fetus* and released in mother's blood (17), has been identified. Although the majority of HERVs lack function due to negative selective pressures and mutations occurred during evolution (15), some HERVs have been coopted for physiological functions (18, 19) while their reactivation has been frequently associated with human pathological conditions.

In previous works we demonstrated a distinctive expression of several HERV families (HERV-H, HERV-K, and HERV-W) in peripheral blood mononuclear cells (PBMCs) from ASD and ADHD children. Particularly, HERV-H was higher in patients than in controls and closely related with the symptomatology, being more expressed in children with severe symptoms (10, 11). Furthermore, in ADHD patients, undergoing methylphenidate therapy, HERV-H expression decreased when the clinical signs regressed (20, 21). Also, in two preclinical ASD models (inbred BTBR T+tf/J and valproate-treated CD1 mice) we demonstrated changes of expression of six ERVs families, proinflammatory cytokines and Toll-Like Receptors in whole embryos, blood, and brain samples (22). Interestingly, in the valproate-induced ASD model the increased expression of ERVs and early behavioral alterations were inherited across generations *via* maternal lineage (23). In the present study, first of all, we analyzed HERV-H, HERV-K, HERV-W, and HEMO expression in PBMCs from ASD patients and their parents, to look for a common molecular trait within family members. Then, as immune deregulation is involved in ASD pathogenesis (24), we assessed the expression of several cytokines in order to evaluate any their possible correlation with HERV expression levels.

## Materials and Methods

### Patients and Healthy Controls

The study included 133 Caucasian individuals, belonging to 45 families, of which 31 with autistic patients (ASD families), and 14 enrolled as healthy controls (HC) (Table 1). No statistical differences were found in the comparison of the median age among the children, mothers, and fathers groups, and for sex ratio between the children groups. Autistic patients and their parents were recruited at the Child Neurology and Psychiatry Unit of “Tor Vergata” Hospital (Rome, Italy). Enrolment started before of the advent of DSM-5 (Diagnostic and Statistical Manual of Mental Disorders, fifth edition), so ASD patients were diagnosed according to the DSM-IV-TR diagnosis criteria as Autistic Disorder (AD), or Pervasive Developmental Disorder-Not Otherwise Specified (PDD-NOS) (Table 2). All ASD children performed an evaluation of symptomatologic profile with Autism Diagnostic Interview-Revised (ADI-R), Autism Diagnostic Observation Schedule (ADOS), including a specific index of symptoms severity calculated with Calibrate Severity Score (CSS). The developmental level was assessed by using the Psycho-educational Profile-Third edition (PEP-3). ASD patients with known infectious, metabolic or genetic diseases, chromosomal abnormalities, seizures, identifiable neurological syndromes, or focal signs were excluded from the study. All children were tested by genetic analysis, and none of them had fragile X syndrome or other chromosomal abnormalities.

**Table 1:** Demographic information of individuals included in the study.

	**ASD**	**Healthy controls**
**Number of families**	31	14
**Children**^**#**^	31	21
Male	28	18
Female	3	3
Ratio Male/Female	9.33	6
Median age (range)^*^	4.33 (2–10)	5 (2–17)
**Mothers**^**#**^	28	14
Median age (range)^*^	35 (29–42)	36.5 (26–43)
**Fathers**^**#**^	29	10
Median age (range)^*^	42 (29–47)	43.5 (29–47)

# Number of individuals.

* *Years*.

**Table 2:** Clinical characteristics of ASD children.

		**Diagnosis**	
		**AD**	**PDD-NOS**
Number of ASD patients	31	26	5
CSS	4.965 (1–10)^*^		
Global DQ	0.686 (0.42–0.97)^*^		

* Mean value (range).

*AD, Autistic Disorder; PDD-NOS, Pervasive Developmental Disorder-Not Otherwise Specified; DQ, Developmental Quotient (calculated by PEP-3 scale); CSS, Calibrate Severity Score*.

All patients were free of drugs at the time of blood collection. Age- and sex-matched healthy volunteers were recruited from personnel of the Faculty of Medicine of “Tor Vergata” University and the Child Neurology and Psychiatry Unit of “Tor Vergata” Hospital (Rome, Italy) and enrolled together with their children who attended outpatient facilities for routine examinations at the same hospital. None of the members of the control groups had a history of neurological, psychiatric, or infectious disorders. All control subjects did not take drugs at the time of the recruitment.

### Samples Preparation, RNA Extraction, and RT-Real Time PCR

Peripheral blood mononuclear cells (PBMCs) from heparinized blood samples of all the individuals enrolled in the study were isolated by density gradient centrifugation (Lympholyte-H, Merck Darmstadt, Germany) and collected immediately after the separation. Pellets were frozen rapidly in liquid nitrogen and stored at −80°C until use (10). Total RNA was extracted from 0.8 × 10^5^ PBMCs using NucleoSpin RNA kit according to the manufacturer's instructions (Macherey-Nagel, Dueren, Germany). Two hundred fifty nanogram of DNase-treated RNA were reverse-transcribed into cDNA using Improm-II Reverse Transcription System (Promega, Fitchburg, WI, USA) according to the manufacturer's protocol, in a total volume of 20 μl. The transcriptional levels of several HERV families and cytokines were assessed by Real-time PCR in the Bio-Rad instrument CFX96 Real-Time System, using SYBR Green chemistry (iTaq Universal SYBR green Supermix, Biorad). Specific pairs of primers for *env* of HERV-H, HERV-K, HERV-W (10), HEMO (17), and for cytokines expression were used (**TNF-α**: Gene Bank Access Number NM_000594.3; forward 5′-CCCGAGTGACAAGCCTGTAG-3′, reverse 5′-TGAGGTACAGGCCCTCTGAT-3′; **IFN-γ**: NM_000619.2; forward 5′-TCAGCTCTGCATCGTTTTGG-3′, reverse 5′-GTTCCATTATCCGCTACATCTGAA-3′; **IL-10**: NM_000572.2; forward 5′-ACATCAAGGCGCATGTGAAC-3′, reverse 5′-CACGGCCTTGCTCTTGTTTT-3′; **IL-8**: NM_000584.3; forward 5′-TCTTGGCAGCCTTCCTGATTT-3′, reverse 5′-TTCTGTGTTGGCGCAGTGT-3′; **IL-1β**: NM_000576.2; forward 5′-CCACCTCCAGGGACAGGATA-3′, reverse 5′-AACACGCAGGACAGGTACAG-3′; **IL-6**: NM_000600.3; forward 5′-TGCAATAACCACCCCTGACC-3′, reverse 5′-ATTTGCCGAAGAGCCCTCAG-3′).

To set up the Real-Time PCR a serial dilution (10-fold) was done to calculate efficiencies and correlation coefficient. The amplification efficiency was calculated by the formula [efficiency = 10(−1/slope)] and all the primer pairs showed an efficiency ranging from 0.95 to 0.97. To verify the specificity of the primers and to exclude any false positives, DNA sequencing of PCR-samples from individuals belonging to ASD and HC families were performed. Real-Time PCR included 0.20 μl of cDNA, 10 μl of SYBR green Supermix, and specific primers ranging from 100 to 200 nM, in a total volume of 20 μl, and was conducted for 1 cycle at 95°C for 5 min and then for 45 cycles of 95°C for 10 s and 60°C for 15 s. Each sample was analyzed in triplicate and to check out any possible contamination, a negative control was included in each experiment. The housekeeping gene β-glucuronidase (GUSB) (10) was used to normalize the results. Each experiment was completed with a melting curve analysis to confirm the specificity of amplification and the lack of non-specific products and primer dimers. Quantification was performed using the threshold cycle (Ct) comparative method and the relative expression was calculated as follows

$$2^{-[\Delta \text{Ct}(\text{sample})-\Delta \text{Ct}(\text{calibrator})}=2^{-\Delta \Delta \text{Ct}}$$

where ΔCt (sample) = [Ct (target gene)—Ct (housekeeping gene)] and ΔCt (calibrator) was the mean of ΔCT of all the samples from healthy control children.

### Statistical Analysis

The Mann Whitney *U*-test was used to compare HERV and cytokines expression levels in PBMCs from children with ASD and their parents and corresponding age- and sex- matched healthy controls (HC). To determine any association between different parameters, we employed multivariate regression models which always included child age, gender as well as diagnosis as nuisance covariates on log-transformed data. The receiver operating characteristic (ROC) curves and the respective areas under the curve (AUCs) with 95%CI were used to assess the sensitivity and specificity of HERV expression for the identification of ASD family members. Statistical analyses were carried out using SPSS software (version 20.0). Statistical significant comparisons were considered when *p* < 0.050.

## Results

### ASD Children and Their Parents Display Peculiar Transcriptional Activity of HERVs

**Figure 1:**
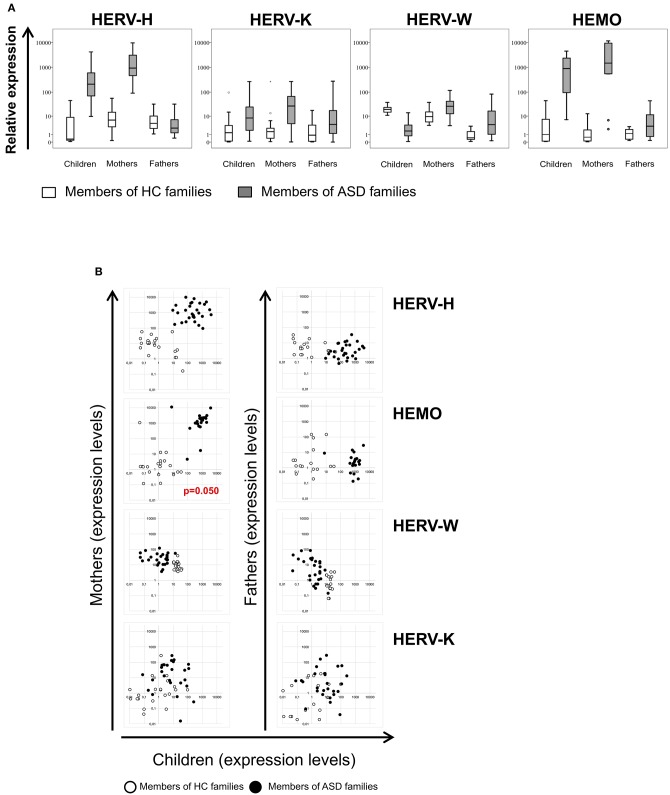
**(A)** Relative expression of HERV-H, HERV-K, HERV-W, and HEMO in PBMCs of patients with ASD and their parents in comparison to healthy control groups. HERV transcriptional activity was evaluated in PBMCs of children, mothers, and fathers of healthy control families (HC families, white) and of children with ASD and their parents (ASD families, gray) by RT-Real time PCR. The results are represented as box plots, depicting mild (black dot), and extreme outliers (asterisk) for each group. **(B)** Scatter plots of HERVs expression in children vs. their parents. Dots depicted in white and black are related to data obtained from HC and ASD families, respectively.

The env gene expression of HERV-H, HERV-K, HERV-W, and HEMO was investigated in peripheral blood mononuclear cells (PBMCs) from ASD children and their parents and from corresponding healthy controls (HC) by quantitative RT-Real time PCR analysis. The obtained data are represented as box plots in Figure 1A and median values and interquartile ranges are reported in Table 3.

**Table 3:** Median values and interquartile range of HERV relative expression in PBMCs from ASD patients, healthy controls and their parents, and in healthy controls.

		**HERV-H**	**HERV-K**	**HERV-W**	**HEMO**
Children	HC	0.39 (0.15–14.18)^*^	3.07 (0.09–7.78)	21.47 (15.75–27.42)	1.37 (0.13–2.97)
	ASD	106.71 (60.02–1082.49)	9.73 (5.31–40.92)	1.28 (0.65–3.35)	865.47 (561.49–1132.35)
Mothers	HC	4.41 (1.50–24.91)	1.62 (0.39–3.50)	11.79 (4.63–15.32)	1.48 (0.68–3.48)
	ASD	746.92 (401.15–2241.05)	9.88 (3.56–78.06)	21.82 (12.38–43.07)	1731.02 (903.16–2182.97)
Fathers	HC	4.66 (2.31–12.12)	0.95 (0.07–6.24)	0.49 (0.28–1.77)	1.32 (1.01–50.43)
	ASD	1.64 (0.80–3.28)	1.38 (1.12–27.88)	7.73 (0.89–23.86)	2.4 (1.4–4.19)

* *Median values (IQR)*.

In PBMCs from autistic patients, HERV-H transcriptional levels were significantly higher respect to children of HC group (*p* < 0.001). Intriguingly, also PBMCs from mothers of children with ASD showed significantly higher levels of HERV-H transcripts respect to mothers of HC group (*p* < 0.001), while no differences were observed comparing fathers of the two groups. Within the families with autistic children (hereafter indicated as ASD families), the transcriptional activity of HERV-H was significantly higher in the mothers (*p* < 0.001), and significantly lower in the fathers (*p* < 0.001) respect to their children, and HERV-H was significantly higher in mothers respect to the fathers (*p* < 0.001). Within the families of healthy control children (hereafter indicated as HC families), HERV-H transcriptional levels were significantly higher in mothers as well as in fathers respect to their children (*p* ≤ 0.020), while in the comparison between mothers, and fathers no significant differences were observed.

The HERV-K transcriptional activity was also significantly higher in PBMCs from children with ASD and their mothers respect to corresponding controls (*p* ≤ 0.003), and no differences were observed comparing the fathers of the two groups. Within ASD families, the HERV-K expression levels were similar in mothers and children, but significantly different between mothers, and fathers (*p* = 0.017). No statistically significant differences were detected between the members of the HC families.

The transcriptional activity of HERV-W resulted instead significantly lower in autistic children respect to controls (*p* < 0.001), and significantly higher in their mothers, and fathers respect to corresponding HC groups (*p* = 0.004 for both the comparisons). Within ASD families the transcriptional activity of HERV-W was lower in children than in their parents (*p* ≤ 0.011) and, among parents, higher in mothers (*p* < 0.001). Conversely, children of HC families showed high expression levels compared with their parents (*p* ≤ 0.008) and, among these, higher levels were observed in mothers (*p* < 0.001).

The transcriptional activity of HEMO was similar to that found for HERV-H; indeed, in PBMCs from both autistic children and their mothers, HEMO expression was significantly higher compared to the corresponding HC (*p* < 0.001 for both the comparisons), while no differences were observed in the comparison between the two groups of fathers. Within ASD families, the transcriptional activity of HEMO was significantly higher in children and their mothers respect to fathers (*p* ≤ 0.002).

No statistically significant differences were observed between the members of the HC families.

### HERV-H and HEMO Activity Is Associated in Children and Their Mothers

**Figure 2:**
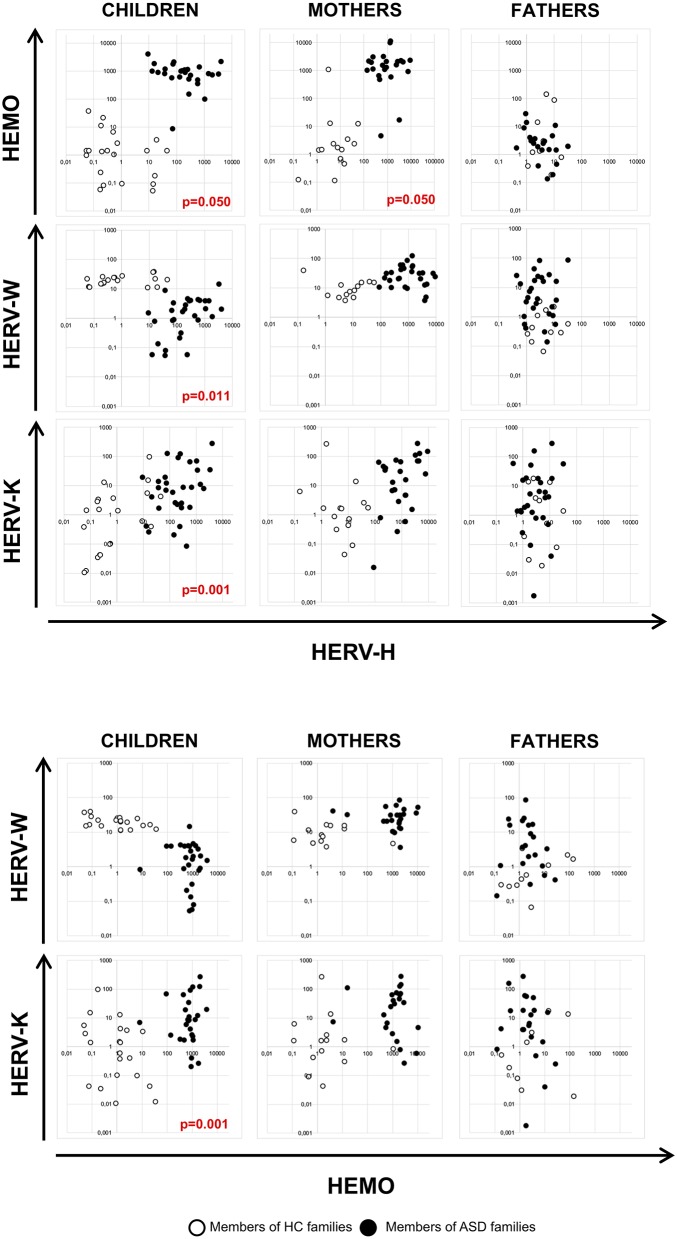
Scatter plots of HERV and HEMO expression levels in patients with ASD and their parents and in healthy control individuals. Dots depicted in white and black for members of HC and ASD families, respectively.

The multivariate regression models, which included child age, gender and diagnosis as nuisance covariates, have been employed to assess any association of HERV transcriptional activity among children and their parents and among HERVs in all the individuals analyzed. The analysis demonstrated that HEMO expression in children was found to be positively associated with HEMO expression in their mothers (*p* = 0.050), irrespective of diagnosis, sex and age (Figure 1B). Moreover, by the plotting of HERV-H and HEMO expression levels of children *vs* their parents, ASD patients and their mothers can be qualitatively distinguished from corresponding members of HC families (Figure 1B), since HERV-H/HEMO were high both in ASD children and their mothers while were low in corresponding HC individuals. Regarding the analysis among HERV expression, a negative association between HERV-H and HEMO in children and their mothers (*p* = 0.050, both for children and mothers) was observed, irrespective of diagnosis, sex, and age. Moreover, in children a positive association between HERV-H and HERV-K (*p* = 0.001), and HERV-H and HERV-W (*p* = 0.011) was found, while HEMO was negatively associated to HERV-K (*p* = 0.037) (Figure 2), all irrespective of diagnosis, sex, and age. No statistically significant association among the expression levels of the different HERVs in fathers was reached, for all the possible matches. Furthermore, only children and their mothers can be qualitatively distinguished as belonging to ASD or HC families by plotting the expression levels of the different HERVs and HEMO. In fact, high levels of the matched HERVs in patients and their mothers correspond to low levels in HC (as in the case of HEMO *vs* HERV-H, of HERV-K *vs* HERV-H, and of HERV-K *vs* HEMO) or *vice versa* (as in the case of HERV-W *vs* HERV-H) (Figure 2).

### Cytokines Expression Is Altered in ASD Children and Their Mothers

**Figure 3:**
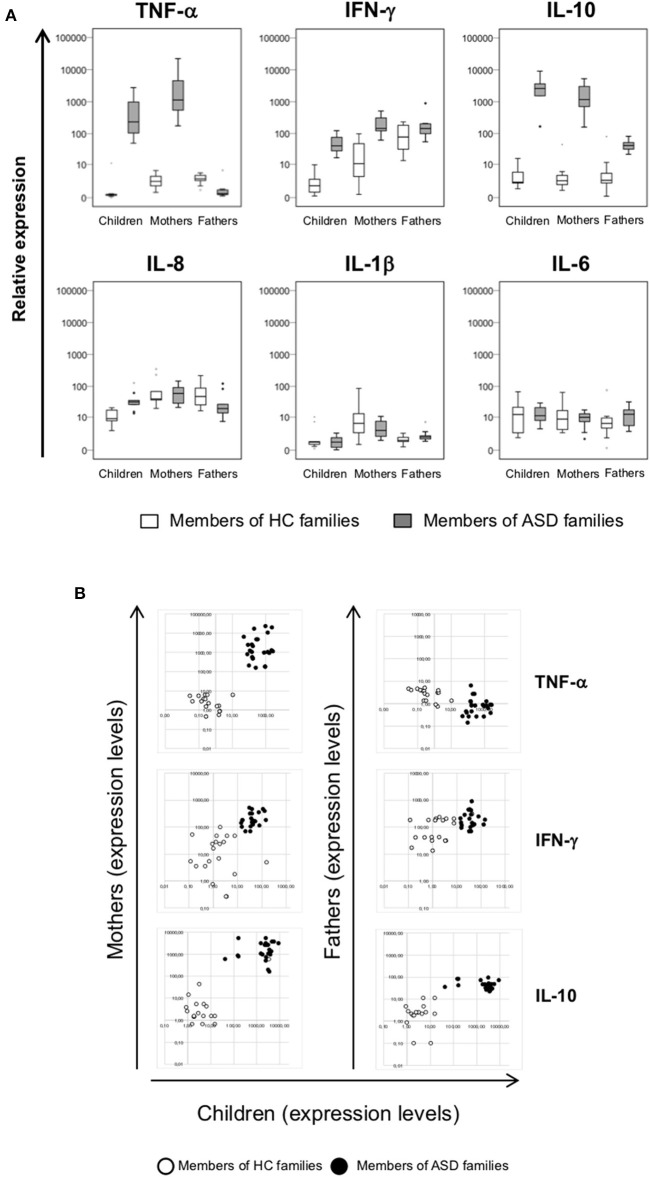
**(A)** Relative expression of TNF-α, IFN-γ, IL-10, IL-8, IL-1β, and IL-6 in PBMCs from patients with ASD and their parents in comparison to healthy control individuals. Cytokines expression levels were analyzed in PBMCs of children, mothers, and fathers of healthy control families (HC families, white) and of children with ASD and their parents (ASD families, gray) by RT-Real time PCR. The results are represented as box plots, depicting mild (black dot) and extreme outliers (asterisk) for each group. **(B)** Scatter plots of cytokines expression in children vs. their parents. Dots depicted in white and black are related to data obtained from HC and ASD families, respectively.

The expression of a selected group of cytokines (TNF-α, IFN-γ, IL-10, IL-8, IL-1β, and IL-6) was analyzed in PBMCs, obtained from individuals belonging to ASD and HC families by RT-Real time PCR assay. Due to the low sample volume of blood collected, only 21 autistic patients, and corresponding parents were analyzed, while all the healthy controls were included. The data are represented as box plots (Figure 3A) and median values and interquartile ranges are reported in Table 4.

**Table 4:** Median values and interquartile range of cytokines relative expression in PBMCs from ASD patients and their parents and in healthy controls.

		**TNF-α**	**INF-γ**	**IL-10**	**IL-8**	**IL-1β**	**IL-6**
Children	HC	0.25 (0.15–0.29)^*^	1.35 (0.40–2.91)	2.02 (1.41–7.95)	8.81 (5.79–17.77)	0.79 (0.43–3.82)	13.32 (2.54–26.24)
	ASD	251.17 (101.04–1153.65)	40.46 (27.62–85.81)	2603.20 (1197.99–3847.43)	31.77 (23.46–42.07)	0.81 (0.20–1.73)	11.22 (6.87–21.04)
Mothers	HC	2.26 (0.97–4.57)	11.38 (2.85–48.91)	2.37 (1.52–4.31)	39.64 (33.66–149.58)	5.99 (2.28–17.66)	8.42 (3.28–23.07)
	ASD	1134.42 (459.67–4946.93)	144.42 (116.04–319.27)	1178.35 (651.34–3023.56)	60.46 (28.40–98.24)	3.19 (1.68–8.32)	9.83 (5.81–13.88)
Fathers	HC	2.94 (2.19–4.16)	90.62 (31.40–186.71)	2.44 (1.36–8.11)	48.92 (24.92–89.66)	1.05 (0.75–1.56)	5.92 (2.70–9.95)
	ASD	0.33 (0.26–0.77)	143.18 (96.82–203.19)	41.46 (28.75–61.57)	19.38 (12.54–54.86)	1.61 (1.24–2.34)	12.44 (4.22–18.95)

* *Median values (IQR)*.

Higher expression levels of TNF-α have been found in PBMCs from autistic patients and their mothers when compared to corresponding controls (*p* < 0.001) and to fathers (*p* < 0.001), while fathers of autistic patients showed not significantly different expression respect to fathers of HC group.

The same trend was highlighted for IFN-γ, whose the expression levels were more elevated both in children and their mothers respect to corresponding controls (*p* < 0.001) while no significant difference were observed between ASD and HC fathers groups. In ASD children and their parents the IL-10 expression levels were higher respect to corresponding HC (*p* ≤ 0.004), with higher values in patients and their mothers respect to fathers (*p* < 0.001). Inversely, IL-8 expression was higher only in autistic patients respect to children of HC families (*p* = 0.001), and no differences were found in all other comparisons. No differences were found for the expression levels of IL-1β and IL-6 in the comparison between members of ASD and HC families. Moreover, by plotting TNF-α, IFN-γ, IL-10 expression levels of children and their parents, ASD patients, and their mothers may be qualitatively distinguished from corresponding members of HC families (Figure 3B), since cytokines expression values were high both in ASD children and their mothers while were low in corresponding HC individuals.

### Analysis of the Association Among HERVs and HEMO Activity With Cytokines Expression

**Figure 4:**
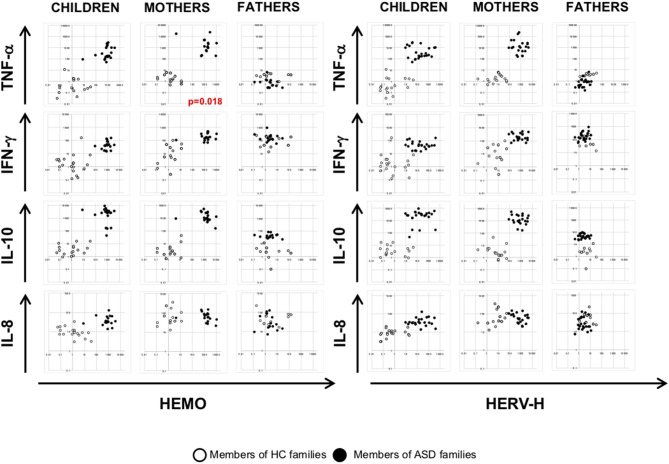
Scatter plots of HERV-H and HEMO vs. cytokines expression levels. Dots depicted in white and black for members of HC and ASD families, respectively.

**Figure 5:**
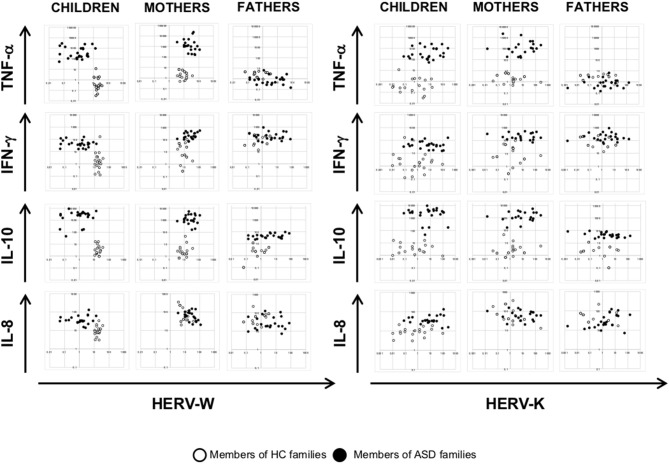
Scatter plots of HERV-W and HERV-K vs. cytokines expression levels. Dots depicted in white and black for members of HC and ASD families, respectively.

To investigate any association among HERV transcriptional activity and cytokines expression, we employed multivariate regression models, including child age, gender, and diagnosis as nuisance covariates. The analysis demonstrated that HEMO activity is negatively associated with TNF-α in mothers (*p* = 0.018) irrespective of diagnosis, sex and age, while no other associations were found in children and fathers (Figures 4, 5). Furthermore, only children and their mothers can be qualitatively distinguished as belonging to ASD or HC families by plotting HERV-H, HEMO, and HERV-W *vs* TNF-α, IFN-γ, and IL-10 expression levels (Figures 4, 5), since high values of HERV expression correspond to high values of cytokines in patients and their mothers while the expression is low in the corresponding control individuals. Conversely, no distinct groups can be visually recognized when HERV-K expression was plotted against cytokines expression (Figure 5).

### Analysis of Diagnostic Accuracy of HERVs and HEMO

**Figure 6:**
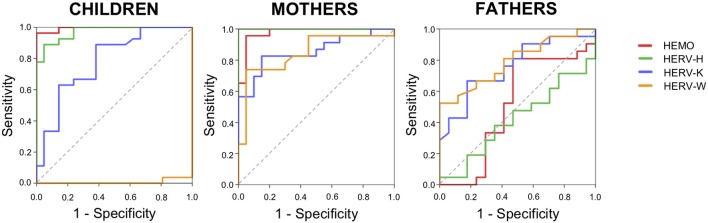
Diagnostic accuracy of HERV expression. Diagrams of ROC curve analysis for HEMO, HERV-H, HERV-K, and HERV-W in children, mothers, and fathers of HC and ASD families.

The results showed significant modification of HERV expression in ASD members, specifically children and mothers, with different patterns in fathers. In order to evaluate the diagnostic accuracy of HERV-H, HEMO, HERV-K, and HERV-W for the identification of ASD family members we performed the analysis of the receiver operating characteristic (ROC) curves and the respective areas under the curve (AUCs) (Figure 6).

The calculated ROC curve area and the value of sensitivity and specificity showed a high diagnostic performance of HERV-H and HEMO for the identification of ASD children and their mothers, but not of their fathers. For all the groups, HERV-K showed high sensitivity but low specificity. In parents, HERV-W showed high sensitivity but a low specificity, while in children HERV-W showed no sensitivity and low specificity (Figure 6) in accordance with the significant downregulation demonstrated in ASD children respect to controls (Figure 1A). Indeed, the ROC analysis to discriminate ASD children showed for HEMO with a cut-off of 20.97 a AUC = 0.995 (95% CI: 0.982–1,000, sensitivity = 92.85%, specificity = 95%), for HERV-H with a cut-off 19.7 an AUC = 0.972 (95% CI: 0.935–1,000, sensitivity = 93.33%, specificity = 86.36%), for HERV-K with a cut-off 14.96 an AUC = 0.782 (95% CI: 0.649–0.916, sensitivity = 83.33%, specificity = 47.5%), and for HERV-W with a cut-off of 36.34 an AUC = 0.007 (95% CI: 0.000–0.023, sensitivity = 0%, specificity = 38%). The ROC analysis to discriminate mothers of ASD children showed for HEMO with a cut-off of 12.48 a AUC = 0.976 (95% CI: 0.937–1,000, sensitivity = 92%, specificity = 95%), for HERV-H with a cut-off 56.6 an AUC = 1,000 (95% CI: 1,000–1,000, sensitivity = 93.33%, specificity = 100%), for HERV-K with a cut-off 13.52 an AUC = 0.857 (95% CI: 0.743–0.971, sensitivity = 88.24%, specificity = 61.29%), and for HERV-W with a cut-off of 16.91 an AUC = 0.847 (95% CI: 0.725–0.969, sensitivity = 88.89%, specificity = 48.72%). The ROC analysis to discriminate fathers of ASD children showed for HEMO with a cut-off of 144.49 a AUC = 0.510 (95% CI: 0.308–711, sensitivity = 0%, specificity = 41.67%), for HERV-H with a cut-off 21.09 an AUC = 0.420 (95% CI: 0.237–0.603, sensitivity = 0%, specificity = 35.56%), for HERV-K with a cut-off 14.35 an AUC = 0.758 (95% CI: 0.604–0.911, sensitivity = 90%, specificity = 44.44%) and for HERV-W with a cut-off of 3.36 an AUC = 0.791 (95% CI: 0.650–0.933, sensitivity = 88.89%, specificity = 53.57%).

## Discussion

In this study we describe for the first time that ASD children and their mothers share common expression levels of HERV-H and HEMO and of the cytokines TNF-α, IFN-γ, and IL-10. The current data highlight that the high HERV-H expression is not an exclusive trait of autistic patients, as we already demonstrated (10, 12), but also of their mothers. Furthermore, HEMO appear to be highly expressed in ASD children and, interestingly, this molecular mark is equally shared by their mothers, suggesting a further common molecular feature between mother and child.

The mother-child association within the ASD families is also supported by the multivariate regression models, highlighting that HEMO expression levels associated children with their mothers. All together, these observations suggest HERV-H, and HEMO expression as common molecular traits in autistic patients and their mothers, in agreement with our previous evidences, obtained from preclinical ASD model induced by prenatally exposure to valproic acid (VPA). In fact, mice from VPA-exposed dams showed high expression of several ERV families from intrauterine life to adulthood (22) and notably, these alterations were inherited *via* maternal lineage across generations, parallel to the autistic-like phenotype (23). Mechanisms by which VPA modifies ERV expression and induces neurobehavioral alteration are still unclear, but since VPA is a non-selective histone deacetylases inhibitor, epigenetic modifications are likely involved.

Moreover, an association between HERV-H and HEMO expression levels in children and mothers but no in fathers was observed. Therefore, on the basis of the expression levels of these two variables, children, and mothers can be distinguished as belonging to ASD or to healthy control families, suggesting that the concomitant altered expression of HERV-H and HEMO is related to ASD. Furthermore, high diagnostic performance for HERV-H and HEMO suggests a potential application of these sequences for the identification of ASD children and their mothers from corresponding controls.

The hypothesis that HERV-H and HEMO are involved in ASD is plausible since several HERV families play important roles in mammalian development and differentiation (17, 25, 26). Indeed, long non-coding RNAs (lncRNAs) by HERV-H elements and the recruitment of specific cellular transcriptional activators on HERV-H long terminal repeats (LTRs) seems to be involved in the conservation of stem cell identity (27–29). Moreover, HERV-H loci seem to be more preserved in a full-length state than other HERV families, suggesting that the full-length elements rather than solo-LTRs are useful to the host and that the internal regions of HERV-H may be involved in the process of exaptation (30). HEMO is an ancestral env gene recently uncovered in humans, expressed in embryos already in the early stages of development and in all subsequent differentiation periods, highly expressed also in the placenta and released in the blood of pregnant women (17).

The events occurring during intrauterine stages are critical for the development of the connections among neurons and among neuronal and glial cells, being potentially responsible of later development of ASD. Considering the functions of HERV-H and the potential role of HEMO in human embryogenesis, their contribution in the derailed neurodevelopmental process as cofactors in ASD etiopathogenesis becomes believable. The involvement of HERVs could be due to their intrinsic responsiveness to external stimuli, translated into the cell as gene expression regulation *via* epigenetic mechanism (7, 31). The epigenetic regulation is essential during embryonic development, when a global remodeling occurs, leading to cell commitment and tissues specification (32) and any alteration could impact on neurodevelopment and cognitive function (33). Therefore, epigenetic modifications could directly link molecular regulatory pathways to environmental factors and potentially explain some aspects of complex disorders like ASD, schizophrenia and ADHD (34). ASD, ADHD, and schizophrenia are all neurodevelopmental disorders characterized by derailed brain development, resulting in brain dysfunction later from childhood to adulthood, sharing vulnerability genes (35), and clinical features (36). It has been shown that the risk of ADHD is higher in ASD families (37) and that ADHD is also the most common comorbidity in autistic patients (38) as well as in the past, ASD has been defined as an early manifestation of schizophrenia since one-third of childhood onset cases received first a diagnosis of ASD (39). From our point of view, these disorders show another common feature regarding altered activity of some HERV families, as demonstrated by our group in peripheral blood mononuclear cells from patients with ASD or ADHD (10–12) and in brain and blood from schizophrenia patients (40, 41).

HERVs, due to their regulatory activity, could affect the expression of coding cellular genes acting as enhancers, and also could modulate the activity of lncRNAs since HERVs are associated with a variety of lncRNA, overlapping in the transcriptional start sites (42). Interestingly, a co-expression of lncRNAs, and autism risk genes in the developing human brain was observed by the analysis on the brain transcriptome (43), suggesting that HERVs may be involved in the altered neurodevelopment and consequently in the pathogenesis of ASD, interfering with several pathways related to the development and function of the nervous system. Moreover, the env protein of HERV-W could contribute to the pathogenesis of schizophrenia acting on the glutamatergic transmission in the brain (44), which results altered also in ASD (45). *In vitro* changes in HERV-W env expression were associated to the modulation of the brain-derived neurotrophic factor (BDNF), neurotrophic tyrosine kinase receptor type 2 (NTRK2), and dopamine receptor D3 (DRD3) (46). BDNF aberrancies have been observed in schizophrenia (47) and ASD (48), suggesting a contribution of abnormal HERV activity in the BDNF-mediated neuroprotective mechanism.

Accordingly with our previous findings HERV-W was found to be differently expressed in affected and healthy children (10, 12) and we actually demonstrated changes of HERV-W activity also in parents. Finally, HERV-K activity was higher in patients than in controls, in contrast with our previous work in Albanian autistic patients (12). This difference could be due to various factors: the diverse ethnicity among the patients, the HERV-K polymorphisms and the variability of HERV-K expression in healthy individuals as well as in patients, probably not related to autism. However, the methodological approach we used didn't allow to analyze all HERV members of a certain family and therefore further studies should be applied to investigated the HERV expression profiles.

In the ASD and HC families, we also evaluated the transcriptional activity of a group of cytokines, selected on the basis of our experience regarding the preclinical studies on ASD mouse models (22) and of the available scientific literature that demonstrated the deregulation of the innate immune response in ASD. The deregulation of the innate and adaptive immune responses can affect brain function and development, pointing out the close interconnection between the immune, and the nervous system. Several potential interactions and mechanisms acting both at systemic and cellular levels (49) in different critical periods from the prenatal to the postnatal phases have been proposed in ASD etiopathogenesis. During the first trimester of pregnancy, the acute immune activation due to maternal viral infection increases the risk for having autistic children (50), and, at the time of birth, elevated levels of IL-1β and IL-4 in mothers have been associated to a subsequent diagnosis of ASD (51). In the postnatal period, several cytokines, and chemokines were associated to the severity of ASD-related symptoms (52, 53). Moreover, high levels of cytokines such as IL-1β, IL-5, IL-6, IL-8, IL- 12, IL-13, IL-17, IL-23, TNF-α, and INF-γ were found in blood and in cerebrospinal fluid of children with ASD compared to healthy controls [see (54) for a comprehensive review].

Our data are in line with the findings reported above, demonstrating high expression levels of TNF-α, IFN-γ, IL-8, and IL-10 in PBMCs from autistic children. High levels of TNF-α, IFN-γ, and IL-10 were found also in mothers of ASD patients, while in their fathers only IL-10 was differently expressed respect to controls. Moreover, by multivariate regression analysis, the association of HEMO activity with TNF-α in mothers was observed, in agreement with the observation in preclinical ASD-mouse model, in which the changes in ERV expression were associated with pro-inflammatory cytokines expression (22).

The obtained data support the hypothesis of an interplay between HERVs and cytokines, but how this could contribute to the derailed neurodevelopment remain an open question. It's known that HERVs could shape innate immune response and several mechanisms have been proposed including the transcriptional regulation of neighboring genes and the stimulation of pattern recognition receptors. The up regulation of HERV transcription could lead to the release of HERV-derived pathogen-associated molecular patterns (PAMPs) that interacting with the sensor of the innate immunity evoke the production of pro-inflammatory effectors such as IFNs, cytokines, and chemokines (59). Since HERVs are physiological expressed in humans (19) they could provide continuous triggers to the host immune sensors. On the other side, the inflammatory effectors induced by HERVs could in turn further increase HERV activity (55–57).

Although the present data support the hypothesis of the involvement of HERVs in ASD, further efforts to achieve definitive proof of these elements as cofactors and not as epiphenomenon of neurodevelopmental disorders are needed. However, HERVs and cytokines could be candidate as molecular signature of ASD that permits to discern affected children and their mothers from healthy controls, highlighting the need to study the family members as a unit. Moreover, considering that ASD is a heterogeneous group of neurodevelopmental disorders, a single determinant alone could not be enough to account for the multifaceted nature of ASD. If our findings will be confirmed in larger studies, the expression of selected HERVs, and cytokines could be considered as ASD-associated biological traits in a set of biomarkers identifiable in pregnant women's blood and potentially useful for an early diagnosis and subsequent intervention.

## Data Availability Statement

The raw data supporting the conclusions of this manuscript will be made available by the authors, without undue reservation, to any qualified researcher.

## Ethics Statement

This study was approved by the University Hospital of Tor Vergata Ethics Committee and all experiments were performed after obtaining signed written informed consent from each parent, in accordance with the Declaration of Helsinki.

## Author Contributions

EB, PC, and PS-V designed the research. EB, CC, CM, AA-D, and IB performed the research. EB, CC, CM, AB, AC, MM, SG, NT, and PC analyzed the data. EB, CC, CM, and PS-V wrote the paper.

### Conflict of Interest

The authors declare that the research was conducted in the absence of any commercial or financial relationships that could be construed as a potential conflict of interest.

## Abbreviations

ASD: Autism Spectrum Disorder
HERVs: Human Endogenous Retroviruses
ADHD: Attention Deficit/Hyperactivity Disorder
HEMO: [human endogenous MER34 (medium-reiteration-frequency-family-34) ORF]
PBMCs: Peripheral Blood Mononuclear Cells
HC: Healthy Controls
DSM-5: Diagnostic and Statistical Manual of Mental Disorders, fifth edition
AD: Autistic Disorder
PDD-NOS: Pervasive Developmental Disorder-Not Otherwise Specified
ADI-R: Autism Diagnostic Interview-Revised
ADOS: Autism Diagnostic Observation Schedule
CSS: Calibrate Severity Score
PEP-3: Psycho-educational Profile-Third edition
GUSB: β-glucuronidase
Ct: threshold cycle
VPA: valproic acid
LTRs: long terminal repeats
PAMPs: Pathogen-associated Molecular Patterns.
